# Teaching medical care in mass casualty incidents through advanced clinical simulation

**DOI:** 10.1186/s41077-026-00459-2

**Published:** 2026-10-27

**Authors:** Alonso Mateos Rodríguez, Emilio Cervera Barba, Ester Mora Bastante, Tomas Villen Villegas, Fernando Neria Serrano, Sophia Denizon Arranz

**Affiliations:** Facultad de Medicina, Universidad Francisco de Vitoria, Madrid, Pozuelo de Alarcon Spain

**Keywords:** Simulation based education, Mass casualty incident, Emergency medical service, Emergency preparedness

## Abstract

**Introduction:**

The threat of intentional mass casualty incidents remains high, making healthcare response a priority. The Hartford Consensus and the THREAT protocol (Threat suppression, Hemorrhage control, Rapid Extrication to safety, Assessment by medical providers, Transport to definitive care) establish recommended actions. A key objective is training civilians as "immediate responders" for hemorrhage control. However, this emergency care training is often limited in medical schools. Francisco de Vitoria University incorporates clinical simulation, including emergency medicine content in the fifth year. This study aims to assess learning outcomes from an activity designed to familiarize students with the Hartford Consensus and THREAT protocol.

**Materials and methods:**

Simulation Activity Development: Students gathered in an enclosed area. Basic principles of action during a terrorist attack were explained. A simulated attack unfolded, requiring students to escape, hide, or fight, and assist victims according to protocol. Wounded individuals were transported to a meeting point for a debriefing, followed by a final group meeting.

**Learning outcome assessment:**

Students completed a questionnaire at the beginning and end of the activity, including technical and subjective questions about perceived preparedness. Statistical analysis used the Chi-square test to compare response frequencies. 112 fifth-year medical students completed the online questionnaire.

**Results:**

Seventy-one pre-activity and 41 post-activity questionnaires were completed. Perceived preparedness significantly improved: initially, 66.2% felt "no" prepared, which decreased to 4.9% after the activity (*p* < 0.001). Correct responses to "first thing to do in an attack" increased from 19.4% to 92.7% (*p* < 0.001). Understanding "improvised means" rose from 85.9% to 100% (*p* = 0.042). No statistically significant differences were observed for more technical questions like hemorrhage or cardiac arrest.

**Discussion:**

Teaching through simulated environments offers undeniable learning benefits. While curriculum integration faces, simulation is a valuable tool in emergency training. Our cost-effective instructional methods proved successful, as shown by survey data. These engaging experiences facilitate knowledge acquisition. Replicating real-life conditions helps students appreciate inherent challenges. We conclude that using a simulated environment at the undergraduate level to train students as first responders in mass casualty incidents is an effective educational strategy.

## Introduction

The threat of intentional mass casualty incidents remains high due to ongoing terrorist activity affecting the majority of countries [1]. The ability to manage such events has been identified as a priority for healthcare services. Recent events reveal opportunities for improvement in the response to these types of incidents. The Hartford Consensus establishes the recommended actions in cases of intentional mass casualty events and active shooter scenarios [2]. The acronym summarizing the Hartford Consensus is THREAT (Threat suppression, Hemorrhage control, Rapid Extrication to safety, Assessment by medical providers, Transport to definitive care).

One of the goals of the Hartford Consensus is to train civilians to provide emergency assistance. During intentional mass casualty events, bystanders present at the scene can often be helpful in performing initial hemorrhage control [3]. Traditionally referred to as "bystanders," these immediate responders should not be seen as passive observers but rather as capable of performing life-saving frontline interventions. Immediate responders play a critical role in eliminating preventable prehospital death by controlling external bleeding.

However, the teaching of this type of emergency care in medical schools is often limited to emergency medicine curricula, which are not universally implemented [4, 5]. There are several publications supporting the use of clinical simulation in this type of incident, including within nursing practice [6, 7].

Francisco de Vitoria University offers a clinical simulation curriculum that includes various competencies throughout all academic years [8]. In the fifth year of the medical degree, there is a subject that incorporates emergency medicine content, using clinical simulation as an instructional tool to meet some of the course objectives [9]. Among these objectives was for students to become familiar with the Hartford Consensus and the THREAT protocol. As part of the simulation, an activity was designed to train students in the management of mass casualty incidents.

The aim of this study is to describe an educational activity focused on medical care in mass casualty incidents for medical students, using an advanced clinical simulation scenario.

## Materials and methods

### Simulation activity development

Students gather in an enclosed area with no possibility of escape. In the first part of the simulation, the basic principles of action during a terrorist attack are explained.

The scenario then unfolds: a terrorist group enters the building and begins attacking the students. The students' objective is to follow the established procedure. Minutes later, instructors dressed as terrorists fire simulated weapons with foam projectiles at students acting as victims. Students must choose to escape, hide, or fight, in that order, as recommended by the protocol. Additionally, they must assist the injured. When the simulation team sees someone has been shot, they provide a paper detailing the injury. If someone attempts to help, they must read the paper and proceed with immediate care.

The wounded must be transported to a meeting point, where a debriefing takes place during a third phase of the session. A descriptive debriefing is performed at the end of the activity.

### Learning outcome assessment

At the beginning and end of the activity, students complete a questionnaire containing technical questions on specific care concepts, as well as subjective questions about their perceived preparedness for such incidents. The questionnaire administered to the students is not validated. It was developed by the course teaching team (Appendix 1).

A statistical analysis using the Chi-square test was conducted, comparing the frequencies of each response category before and after the activity.

The questionnaire was completed online via Google Forms by 112 fifth-year medical students.

## Results

A total of 71 questionnaires were completed before the activity and 41 afterward.

**Fig. 1:**
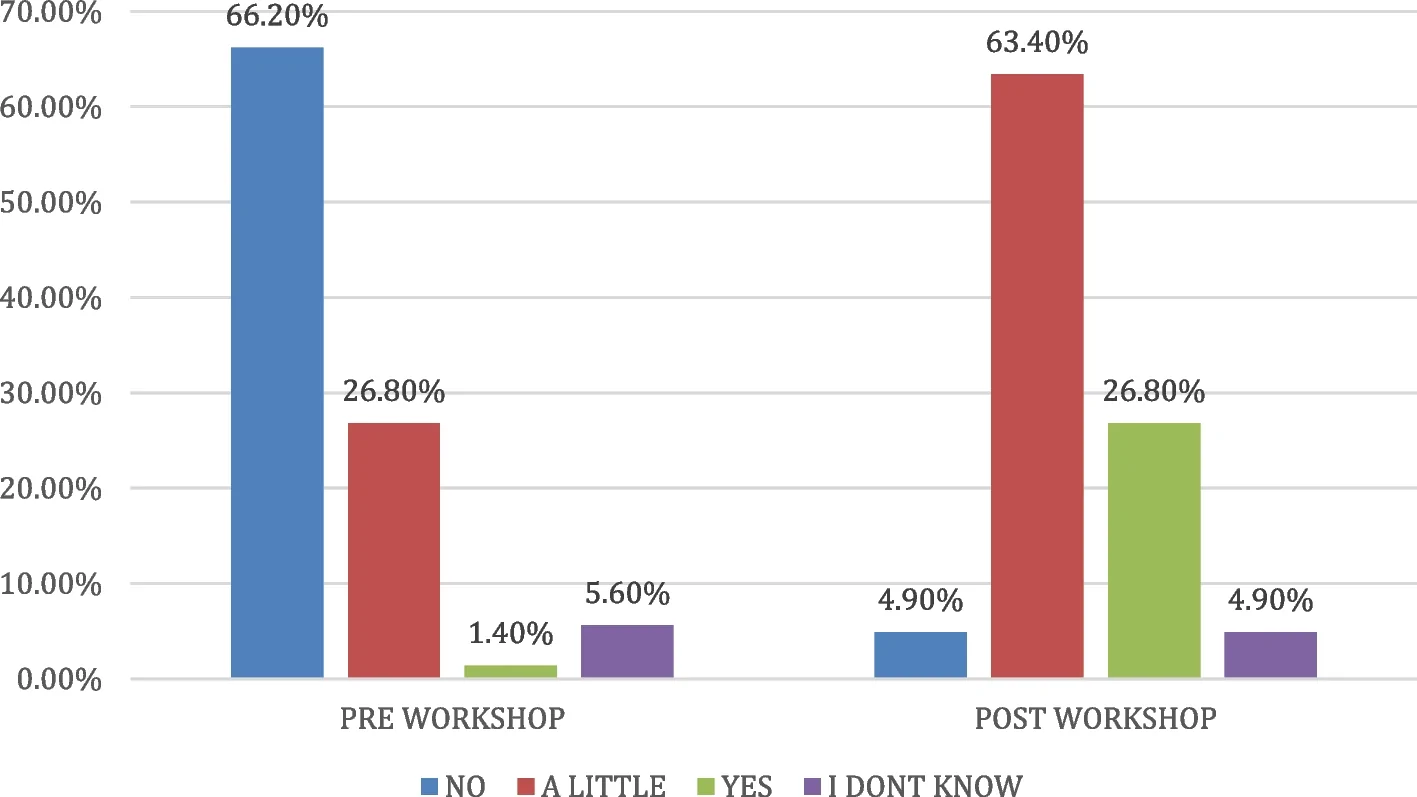
Frequencies of pre- and post-workshop responses to the question; Do you feel prepared to know how to act in a multi-victim incident?

Regarding participants’ perceived preparedness (Question 1), a notable shift was observed following the training intervention. Prior to the workshop, most participants reported not feeling prepared (66.2%), while only 1.4% considered themselves prepared. After the intervention, this distribution changed substantially: the proportion of participants who felt prepared increased to 26.8%, and those who reported feeling “only a little” prepared rose to 63.4%, whereas negative responses decreased to 4.9%. These differences were statistically significant (*p* < 0.001), indicating a clear improvement in participants’ perceived preparedness after the intervention (Fig. 1).

In Question 2, although there was a shift toward more correct responses after the intervention, these differences were statistically significant (*p* < 0.05), suggesting that the workshop produce a measurable change for this specific item.

For the remaining variables, changes were observed between pre- and post-workshop responses; however, most of these differences did not reach statistical significance (Table 1).

**Table 1:** Data questionary

Question	Pre-workshop	Post-workshop	*p*-value
Do you feel prepared to know how to act in a multiple-casualty incident?			***p* < 0.001**
No	47 (66.2%)	2 (4.9%)	
Only a little	19 (26.8%)	26 (63.4%)	
Yes	1 (1.4%)	11 (26.8%)	
Don't know/No answer	4 (5.6%)	2 (4.9%)	
What do you think is the first thing to do if you find yourself in an attack?			***p* < 0.001**
Run Away	14 (19.7%)	38 (92.7%)	
Hide	19 (26.8%)	1 (2.4%)	
Call 112	38 (53.5%)	2 (4.9%)	
When calling 112 (Emergency Services). I must provide the following information:			*p* = 0.479
All the information	65(91.5%)	69(95.1%)	
Incident place	6 (8.5%)	2 (4.9%)	
What is defined as "makeshift means"?			*p* = 0.042
Any item that can be used for therapeutic purposes	61 (85.9%)	41 (100%)	
The medical devices you happen to have availabl	4 (5.6%)	0	
None above	6 (8.5%)	0	
In cases of chest wounds. which is the most correct action?			*p* = 0.006
Pack the wound	36 (50.7%)	12(29.3%)	
Apply a chest seal	1 (1.4%)	7 (17.1%)	
Both above	25 (35.2%)	18 (43.9%)	
None above	9 (12.7%)	4 (9.8%)	
In cases of limb hemorrhage. point out the correct option:			*p* = 0.047
It is advisable to keep the limb elevated	38 (27%)	16 (40%)	
The vessels must be clamped	26 (36.6%)	22 (55%)	
A tourniquet is not recommended	17 (23.9%)	2 (5%)	
It can be left untreated; it is not a life-threatening hemorrhage	1 (1.4%)	0	
In wounds with significant loss of substance. point out the correct option:			*p* = 0.125
We can only use a tourniquet	14 (19.7%)	4 (10%)	
Packing is advisable	46 (64.8%)	34 (85%)	
They cannot be managed with improvised means	8 (11.3%)	2 (5%)	
We will never use improvised means	3 (4.2%)	0	
In caring for victims of an attack. special care must be taken in:			*p* = 0.278
Avoid hypothermia	69 (97.2%)	41 (100%)	
Avoid contact with the victims	2 (2.8%)	0	
Life-saving maneuvers in victims of attacks consist of:			*p* = 0.683
Any maneuver intended to save a life	43 (60.6%)	25 (61%)	
Logistics maneuvers by emergency services	7 (9.9%)	6 (14.6%)	
Airway opening maneuvers	21 (29.6%)	10 (24.4%)	
In a terrorist attack with multiple victims. if you find a patient in CPR:			*p* = 0.058
In a multiple-victim incident, CPR is not started	53 (74.6%)	39 (95.1%)	
CPR is started	10 (14.1%)	1 (2.4%)	
Special care must be taken with cervical stabilization	7 (9.9%)	1 (2.4%)	
We begin maneuvers suspecting hypovolemic cause	1 (1.4%)	0	

Similarly, in Question 3 and Question 4, descriptive improvements were noted in the distribution of responses following the training, with a higher proportion of participants selecting appropriate answers post-intervention. Nevertheless, these differences were not statistically significant (*p* > 0.05).

In Question 5 and Question 6, a comparable pattern was observed. While post-workshop responses indicated a trend toward improved knowledge or decision-making, the differences did not reach statistical significance (*p* > 0.05), indicating that these changes could be due to chance.

For Question 7, participants showed an increase in correct responses after the workshop (e.g., greater recognition of appropriate management strategies such as wound packing). However, this improvement was not statistically significant (*p* = 0.125).

In Question 8, nearly all participants already demonstrated correct knowledge in the pre-workshop phase (97.2%), which increased slightly to 100% post-intervention. This ceiling effect likely explains the lack of statistically significant differences (*p* = 0.278).

Question 9 also did not show statistically significant changes (*p* = 0.683), despite minor variations in response distribution after the intervention.

Finally, in Question 10, there was a notable increase in the proportion of correct responses (from 74.6% to 95.1%), suggesting a potentially meaningful improvement. However, this difference approached but did not reach statistical significance (*p* = 0.058).

## Discussion

Overall, the data indicate that the workshop had a positive effect on participants’ knowledge, especially in the more practical and operational aspects of the response to attacks and mass-casualty incidents. The most consistent improvements were seen in self-perceived preparedness, the initial response to an attack, the concept of improvised means, and the management of certain traumatic injuries. At the same time, some items showed a high baseline level of knowledge or limited room for improvement, which may be due either to the participants’ initial level or to the wording of certain questions. In general terms, the results support the usefulness of the training intervention and suggest that its content helps strengthen key competencies in the health response to critical situations.

Teaching through simulated environments yields undeniable learning benefits for students [10]. However, integrating these objectives into the curricula of different medical faculties is still ongoing, particularly due to the high cost of simulation equipment [11] and the need for a large number of personnel trained specifically in this methodology.

In emergency and critical care training, simulation has always been a valuable tool for both reinforcing and acquiring new knowledge. While the literature includes some examples of simulation in mass casualty training using virtual reality, the effectiveness of these and other tools remains to be fully demonstrated [12]. In our case, the instructional methods, in addition to being cost-effective, proved successful, as evidenced by the survey data before and after the activity. These educational experiences, which are also enjoyable and engaging, facilitate knowledge acquisition. Furthermore, since such incidents are fortunately rare, training must necessarily occur in simulated environments, making repeated practice desirable [13]. Replicating the conditions of these real-life situations allows students to appreciate the inherent challenges of providing care in such contexts [14].

We conclude that using a simulated environment at the undergraduate level to train students as first responders in mass casualty incidents is an effective educational strategy.

## Data Availability

No datasets were generated or analysed during the current study.

## Appendix 1

**Table:**

1	Do you feel prepared to know how to act in a multiple-casualty incident?
2	What do you think is the first thing to do if you find yourself in an attack?
3	When calling 112 (Emergency Services), I must provide the following information:
4	What is defined as "makeshift means"?
5	In cases of chest wounds, which is the most correct action?
6	In cases of limb hemorrhage, point out the correct option:
7	In wounds with significant loss of substance, point out the correct option:
8	In caring for victims of an attack, special care must be taken in:
9	Life-saving maneuvers in victims of attacks consist of:
10	In a terrorist attack with multiple victims, if you find a patient in CPR:
